# Palmprint recognition based on gating mechanism and adaptive feature fusion

**DOI:** 10.3389/fnbot.2023.1203962

**Published:** 2023-05-26

**Authors:** Kaibi Zhang, Guofeng Xu, Ye Kelly Jin, Guanqiu Qi, Xun Yang, Litao Bai

**Affiliations:** ^1^School of Automation, Chongqing University of Posts and Telecommunications, Chongqing, China; ^2^Department of Integrated Chinese and Western Medicine, The Second Affiliated Hospital of Chongqing Medical University, Chongqing, China; ^3^College of Business and Economics, California State University, Los Angeles, CA, United States; ^4^Double Deuce Sports, Bowling Green, KY, United States; ^5^Computer Information Systems Department, State University of New York at Buffalo State, Buffalo, NY, United States; ^6^China Merchants Chongqing Communications Research and Design Institute Co., Ltd., Chongqing, China

**Keywords:** palmprint recognition, convolutional neural networks (CNN), gate control mechanism, adaptive feature fusion, deep learning-based artificial neural networks

## Abstract

As a type of biometric recognition, palmprint recognition uses unique discriminative features on the palm of a person to identify his/her identity. It has attracted much attention because of its advantages of contactlessness, stability, and security. Recently, many palmprint recognition methods based on convolutional neural networks (CNN) have been proposed in academia. Convolutional neural networks are limited by the size of the convolutional kernel and lack the ability to extract global information of palmprints. This paper proposes a framework based on the integration of CNN and Transformer-GLGAnet for palmprint recognition, which can take advantage of CNN's local information extraction and Transformer's global modeling capabilities. A gating mechanism and an adaptive feature fusion module are also designed for palmprint feature extraction. The gating mechanism filters features by a feature selection algorithm and the adaptive feature fusion module fuses them with the features extracted by the backbone network. Through extensive experiments on two datasets, the experimental results show that the recognition accuracy is 98.5% for 12,000 palmprints in the Tongji University dataset and 99.5% for 600 palmprints in the Hong Kong Polytechnic University dataset. This demonstrates that the proposed method outperforms existing methods in the correctness of both palmprint recognition tasks. The source codes will be available on https://github.com/Ywatery/GLnet.git.

## 1. Introduction

With the rapid development of information security, various identification technologies using human-specific biometric features have attracted widespread attention. Human-specific biometric features include face, gait, and fingerprints (Shi et al., 2020; Yang, 2020; Zhang et al., 2021). As an emerging recognition technique of biometric features, palmprint recognition can analyze various recognizable features, including main lines, ridges, minutiae, and textures (Zhang et al., 2018). Palmprint features are considered to be unique biometric information for each person, with a high degree of security and reliability. The research on palmprint recognition techniques is also receiving more and more attention from academia (Wang et al., 2019; Zhong et al., 2019; Fei et al., 2020a). Traditional palmprint recognition methods are mainly developed based on both texture an structure features (Fei et al., 2018). Many effective methods have been applied, such as apparent latent direction code (ALDC; Fei et al., 2019) and joint multi-view feature learning (JMvFL; Fei et al., 2020b), have been applied to palmprint recognition. Some researchers also proposed techniques such as 2D palmprint recognition (Korichi et al., 2021) and 3D palmprint recognition (Yang et al., 2020) for different palmprint morphologies. Recently, new breakthroughs in palmprint recognition performance have been achieved by using deep learning methods (Ungureanu et al., 2020). Deep learning-based palmprint recognition methods can avoid information loss caused by traditional manual features and significantly improve recognition performance (Zhong et al., 2019). For example, Liu et al. (2021) proposed a novel and effective end-to-end algorithm for palmprint recognition, known as the similarity metric hash network.

However, most of the current deep learning-based palmprint recognition methods have certain shortcomings. First, general image classification is applied to recognize palmprints (Zhong and Zhu, 2019), which is not specially designed for palmprint features. In other words, the relationship between palmprint features involving fine details, texture and global mainline information is not considered. Some researchers designed specialized palmprint recognition neural networks based on the characteristics of palmprints (Genovese et al., 2019). Most of these specially designed neural networks are developed based on CNN. Affected by the size of the convolution kernel, CNN can only extract local features of palmprints in a limited way, and cannot extract global feature information of palmprints. For palmprint recognition, it is necessary to study the neural network design of palmprint global feature information extraction. The Transformer, a framework based entirely on a self-attentive mechanism with powerful modeling capabilities for global information, has been widely used in image feature extraction (Zhu et al., 2022, 2023; He et al., 2023).

This paper designs a network structure GLGAnet based on CNN and Transformer. CNN is used to extract local features such as texture and fine details of the palmprint. The Transformer module is used to establish the connection between main lines and ridges in the palmprint features. In the network structure, the features extracted by each component in the GLGAnet backbone network are controlled by the designed gating mechanism. The proposed network structure is able to extract global features such as main lines and ridges of the palmprint, local features such as palm texture and fine details. This paper has three main contributions as follows.

1. A new lightweight network-GLGANet is designed to process local features (e.g., texture) and global features (e.g., mainline) of palmprints. The proposed network extracts local features of palmprints by deep convolutional layers and global feature information of palmprints by using Transformer.

2. A gating mechanism is designed based on the different features extracted by the deep convolutional layer and the Transformer module. The proposed gating mechanism first selects the features of the palmprint by using a feature selection algorithm, and then multiplies them with the features extracted by the backbone network to achieve control over the features extracted by the backbone network.

3. An adaptive convolutional fusion module is designed. This module performs dimensionality reduction and fusion of multi-level features extracted by the gating mechanism through multi-level convolution and single convolution.

The rest of this paper is organized as follows. Section 2 briefly introduces the related topics. Section 3 elaborates on the deep learning network structure we proposed for palmprint recognition. Section 4 presents the experimental results. Section 5 offers the concluding remarks.

## 2. Related work

### 2.1. Traditional palmprint recognition methods

After more than two decades of development, traditional palmprint recognition methods have been developed based on hand-crafted and machine learning methods. The most typical hand-crafted feature recognition methods include Competitive code (Kong and Zhang, 2004), Palm code (Zhang et al., 2003), Fusion code (Kong et al., 2006), RLOC (Jia et al., 2008), and BOCV (Guo et al., 2009). These methods are characterized by fast recognition, and a distance measure is often used in the matching process, which may cause a decrease in the correct rate. For example, the competitive coding scheme (CompCode) proposed by Kong and Zhang (2004) uses several Gabor filters in different directions to encode the palmprint features and applies the Hamming distance to match the palmprint. Traditional machine learning recognition methods typically include PCA (Wang et al., 2022), SVM (Yaddaden and Parent, 2022), histogram (Jia et al., 2013), etc. Compared with hand-crafted feature recognition-based methods, these methods are stabler, which extract more palmprint features with a subsequent increase in computational complexity. For example, Patil et al. (2015) extracted palmprint features by some discrete transforms, dimensionalized features using principal component analysis (PCA) and finally used the dimensionalized features for matching.

### 2.2. Deep learning based palmprint recognition methods

In recent years, CNN-based approaches have achieved remarkable performance on many computer vision tasks. Many deep learning-based recognition methods have also been proposed in the field of palmprint recognition. For example, Tarawneh et al. (2018) first used a pre-trained deep neural network VGG-19 (Simonyan and Zisserman, 2014) to extract palmprint features and then used SVM to match and classify palmprints. Dian and Dongmei (2016) used AlexNet (Krizhevsky et al., 2017) to extract palmprint and then combined hausdorff distance to match and recognize palmprint images. Yang et al. (2017) proposed a deep learning and local coding approach. Zhang et al. (2018) proposed an improved Inception-ResNet-v1 network structure and introduced a central loss function for feature extraction PalmRCNN. Matkowski et al. (2019) proposed an end-to-end palmprint recognition network (EE-PRnet). The network consists of two main networks, the ROI localization and alignment network (ROI-lanet) and the Feature Extraction and Recognition Network (FERnet). Most of these deep learning-based palmprint recognition methods are designed for image classification tasks. But there is no design that studies the characteristics of the main lines, fine details, and textures of palmprints. Other researchers have studied the characteristics of palmprints and have integrated deep learning into traditional hand-based design methods. As a typical approach, Genovese et al. (2019) proposed an unsupervised model-PalmNet-which trains palmprint-specific filters through an unsupervised process based on Gabor response and principal component analysis (PCA). The proposed method was experimented on several datasets and achieved remarkable results. Also, Liang et al. (2021) proposed a competitive convolutional neural network (CompNet) with constrained learnable Gabor filters, which extracts palmprint features for recognition by training learnable Gabor convolutional kernels. In addition, some other researchers have considered making neural networks for lightweight palmprint recognition after considering the extent to which palmprint functionality can be run on edge devices. For example, Jia et al. (2022) proposed a lightweight palmprint recognition neural network (EEPNet), which incorporates five strategies such as image stitching and image downscaling to improve palmprint recognition performance. These deep learning-based methods mentioned above are limited by the convolutional kernel perceptual field when extracting features and cannot effectively extract the global contextual information of palmprints, and can only achieve matching by extracting local features for recognition. The proposed GLGAnet integrating CNN and Transformer architecture can not only model the relationship between main lines and ridges of palmprints, but also effectively extract texture information of palmprints.

## 3. The proposed method

In this section first introduces the general framework of the improved network architecture integrating CNN and Transformer. The functions of the individual modules are then described.

### 3.1. Network structure

**Figure 1:**
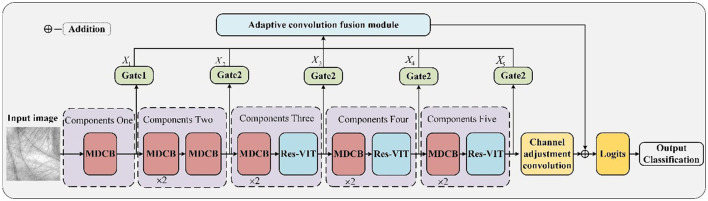
The structure of the proposed network. The *X*_*i*_ expressed the output of the gating mechanism.

The proposed palmprint recognition framework is shown in Figure 1, which mainly consists of a multilayer depth convolution module, a residual Vision Transformer module, a gating mechanism module, and an adaptive convolutional fusion module. The multi-layer depth convolution module is composed of depth-separable convolutional layers, which extract various local features of palmprints, such as ridge features and texture features, from the original palmprint image. The residual VIT module uses Transformer as the backbone structure to model the palmprint image globally and extract global contextual features of the palmprint, such as the orientation features of the three main lines in the palmprint. The gating mechanism module is dominated by the feature selection algorithm, which controls features extracted by the multilayer depth convolution module and the residual VIT module through the feature selection algorithm. The adaptive convolution fusion module performs feature fusion through the gate mechanism of multi-convolution and single convolution.

### 3.2. Multilayer deep convolution block and res-VIT block

Multiple multi-layer depth convolution modules (MDCB) are used to extract local information from palmprints. The multi-layer depth convolution module consists of a depth-separable convolution layer, two normal convolution layers, a regularization layer and two activation layers. The depth-separable convolution learns the correlation of spatial features across channels of the palmprint image, such as local textures of the palmprint. Specifically, when the multilayer deep convolution module receives the input palmpArint image, it first expands the feature volume to multiple channels by ordinary convolution to obtain the output features. Further, the features obtained by ordinary convolution are output to a depth-separable convolution layer to obtain more local feature information of the palm print in a recursive manner across multiple depth-separable convolution layers. The feature volume is then compressed by ordinary convolution layers to the same channel as the input features. Finally, to avoid gradient disappearance during the computation, the feature are superimposed on the original input to obtain the depth convolution feature output *F*_*map*_:


(1) $$\begin{array}{l} \;F_{map}=X+DWConv\left(X\right) \end{array}$$

Where the *X* is the input of the multi-layer depth convolution module.

The most important structure in Res-VIT is the Visual Transformer. The Transformer is developed entirely based on a self-attentive mechanism and has good global modeling capabilities. The input *y* is linearized as *Q, K, andV*, denote key, value, and dimension, respectively. The self-attentive mechanism can be obtained by Equation (2):


(2) $$\begin{array}{l} \text{ Atten}tion\left(Q,K,V\right)=\text{Soft}\max\left(\frac{QK^{T}}{\sqrt{d_{k}}}\right)V \end{array}$$

Since the Transformer structure is designed based on a fully self-attentive mechanism, it lacks the inductive bias in convolution. Therefore, in the Res-VIT module, the input *Y* is first expanded into *M* non-overlapping flat blocks $Y_{U}\in {\mathbb{R}}^{P\times M\times d}$, where *P* = *wh*, and $M=\frac{HW}{P}$, and then encoded between the blocks by applying the Transformer to obtain $Y_{G}\in {\mathbb{R}}^{P\times M\times d}$ as:


(3) $$\begin{array}{l} \;Y_{G}\left(p\right)=\;\times \lambda \text{Transformer}\left(Y_{U}\left(p\right)\right),1\leq p\leq P \end{array}$$

Where λ is the number of Transformer blocks.

After encoding, Res-VIT loses neither the order of the individual blocks nor the order of the pixel space of the palmprint image within each block. Thus, by a collapse operation, *Y*_*G*_ is transformed to *Y*_*U*_, and *Y*_*U*_ is then projected into the lower dimensional space by point-by-point convolution to obtain the features.Finally, the input features of the original palmprint are spliced with this feature by means of the channel adaptive convolution module.

### 3.3. Gate control mechanism

**Figure 2:**
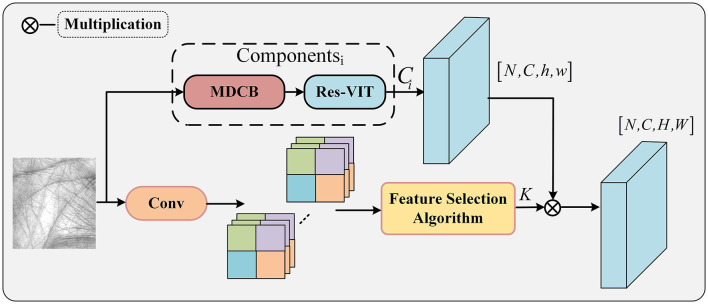
Diagram of the gating mechanism.

The multi-layer depth convolution module extracts local feature information of the palmprint, and Res-VIT extracts global feature information of the palmprint. A gating mechanism is designed to process the extracted information by the convolution and feature selection algorithm. Its structure is schematically shown in Figure 2. Specifically, the palmprint image is first convolved to preliminary palmprint features, and then these features are processed by a feature selection algorithm to select more prominent features of the palmprint. These features are then multiplied with the features extracted from the multi-layer depth convolution and Res-VIT structures, allowing the features extracted from the multi-layer depth convolution and Res-VIT structures to be enhanced.

Feature selection algorithm: First, each input palmprint image is convolved, and then the average value of the input features is calculated. Second, the features in each feature image pixel that are higher than the mean of the feature image pixel are retained and the features that are lower than the mean of the feature image pixel are set to zero to obtain a feature map vector. Finally, this feature map is multiplied with component *i* to control the features obtained from component *i* organization so that the features obtained from component *i* are more prominent. The convolution kernel is updated once for each execution of the feature selection algorithm. The algorithm flow is shown in Algorithm 1.

**Algorithm 1:** Feature selection algorithm.

**Input:**Original image *Y*, control matrix *X*, original image size N*N, cascade output *X*
**Output:**Eigenvector G
1:Function:Feature selection(Y,K,N)
2:initial X=0 and N=0;
3:compute component feature *C*_*i*_ = *conponent*_*i*_(*Y*)
4:compute Original feature mean $\bar{X}=\frac{1}{N*N}\sum Conv\left(Y\right)$
5:**while** *i*<N*N **do**
6: **for** *j* = 1, *j*<N*N; *j*++ **do**
7:**if** $C_{i}<\bar{X}$ **then**
8: *C*_*i*_ = 0;
9: **else**
10: *C*_*i*_ = *C*_*i*_
11: **end if**
12: *G*_*i*_ = *C*_*i*_
13:Updated convolution kernel for (4);
14:**end while**
15: return *G*_*i*_

### 3.4. Adaptive convolution fusion module

**Figure 3:**
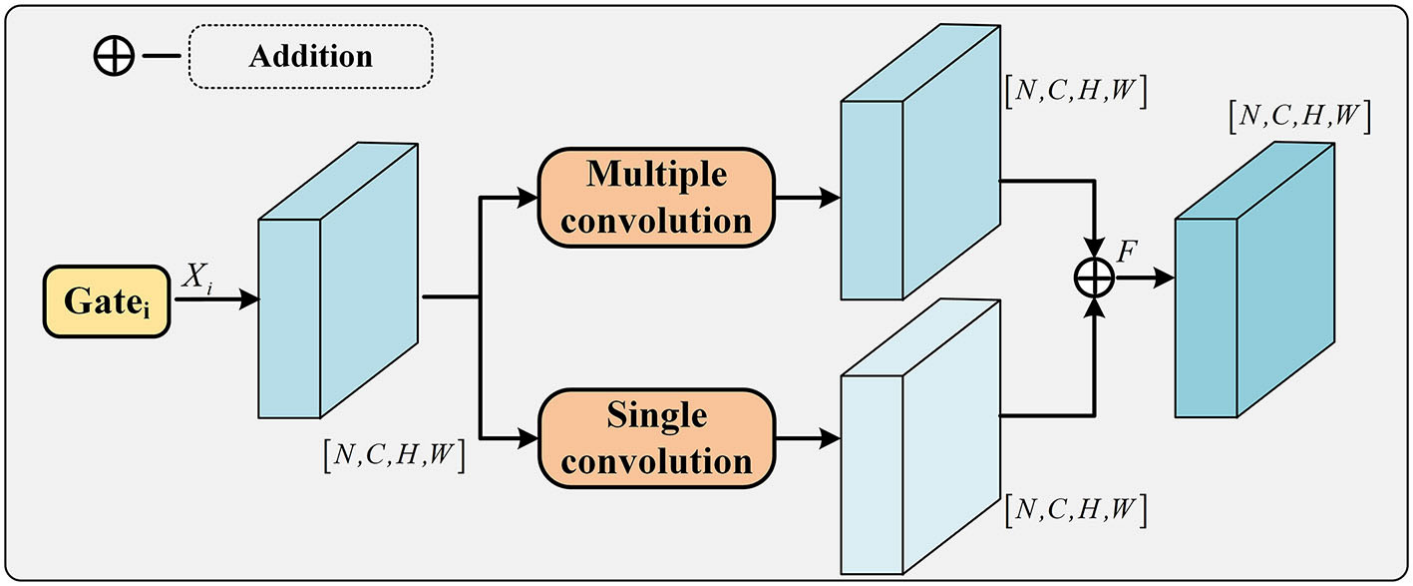
Adaptive convolution module architecture diagram.

The feature maps extracted by the gating mechanism are too large to be effectively fused with the feature maps of the backbone network, and an adaptive convolutional fusion module is designed. A schematic diagram of the adaptive convolutional fusion module is shown in Figure 3. The adaptive convolution fusion module consists of a combination of multi-level convolution and single-level convolution. The fusion module efficiently fuses the feature maps of each level from the output of the gating mechanism with the feature maps extracted from the backbone network. The single-level convolution consists of a convolutional layer, a combined pooling layer and an activation layer. Specifically, in Figure 2, the output of each layer is finally fed into the adaptive convolution module by the gating mechanism, and the first two layers of features can be resized by multi-layer convolution in the adaptive convolution module. Further extraction of local features is also performed. The last three layers have Res-VIT, which extracts the global contextual information of the palmprint, compresses the extracted information by single layer convolution in the adaptive convolution layer, and finally fuses the features of the five layers. The process can be represented by Equation (4).


(4) $$\begin{array}{l} \;F=M\text{ul}tipleConv\left(X_{1,2,3}\right)+\text{Sin}gleConv\left(X_{4,5}\right) \end{array}$$

Where *X*_*i*_ denotes the features output by the gating mechanism and *F* denotes the output features of the adaptive convolution module.

### 3.5. Loss function

Palmprint recognition is essentially a classification problem. To enable the network to achieve good recognition performance, the proposed method is optimized during training in this paper using a cross-entropy loss function, which can be expressed by Equation (5).


(5) $$\begin{array}{l} \;Loss=-\frac{1}{N}\underset{i}{\sum }\overset{M}{\underset{j=1}{\sum }}y_{ij}\log\left({ŷ}_{ij}\right) \end{array}$$

Where *M* denotes the number of categories of palmprints; *y*_*ij*_is a signed function (0 or 1) that takes 1 if the true category of palmprint sample *i* is equal to *j* and 0 otherwise; *y*_*ij*_ denotes the predicted probability that the observed palmprint sample *i* belongs to sample *j*.

## 4. Experiments

### 4.1. Experimental preparation

**Figure 4:**
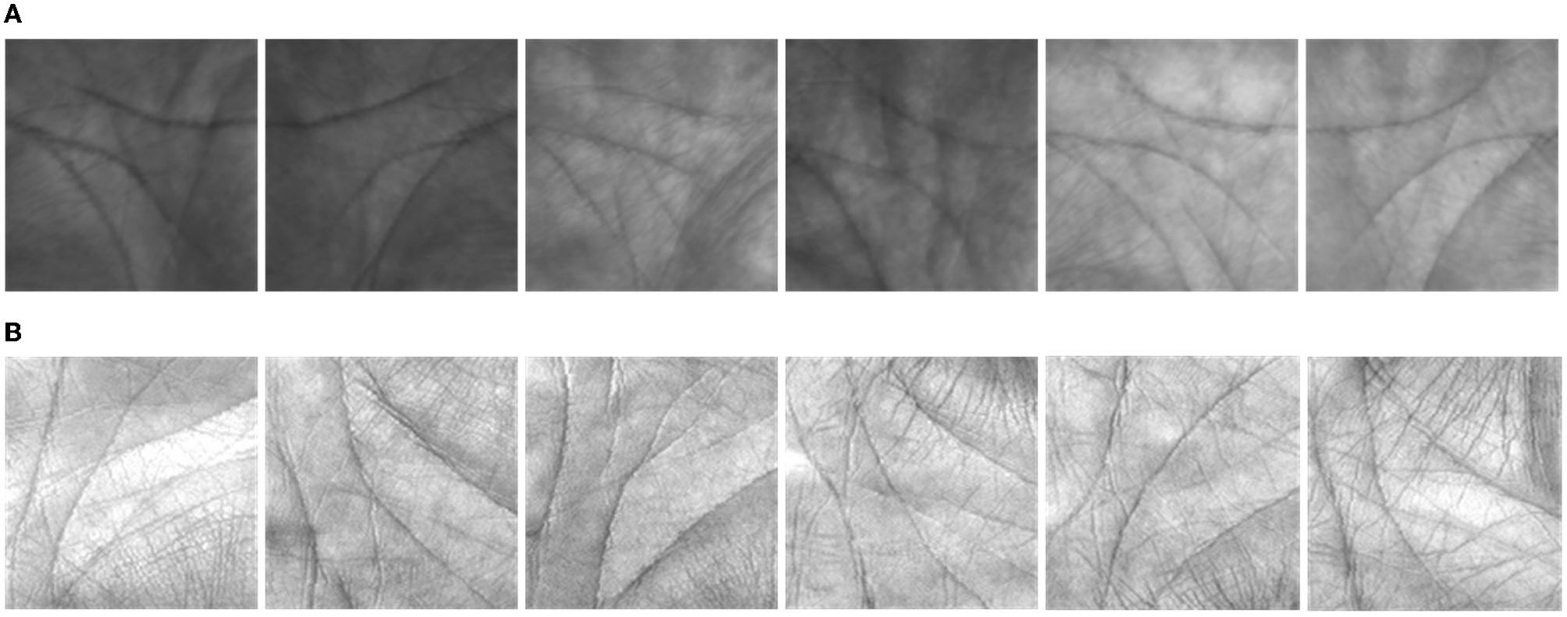
Schematic diagram of the two palmprint datasets. **(A)** Tongji palmprint dataset. **(B)** Poly-U palmprint dataset.

To verify the validity of the proposed method, experiments were conducted on the Tongji University palmprint dataset (Tongji), and the Hong Kong Polytechnic University palmprint dataset (Poly-U). The Tongji dataset collected contactless palmprint images from 300 volunteers, including 192 males and 108 females. All volunteers were from Tongji University in Shanghai, China. There were two collections in total. The average time interval between the first and second acquisition collections was ~2 months. In each collection, 10 palmprint images were collected from each palm of each volunteer, and 6,000 palmprint images were collected from 600 palms. A total of 12,000 (300 × 2 × 10 × 2) high quality non-contact palmprint images were collected in the Tongji dataset. A schematic diagram of the Tongji palmprint dataset is shown in Figure 4A. The Poly-U dataset collected contact palmprint images from 50 volunteers on two occasions. The Poly-U palmprint dataset is shown in Figure 4B.

The proposed model framework was implemented in the Pytorch environment. The input Tongji dataset has an image size of 128*128 and a batch size of 8. The input Poly-U dataset has an image size of 128*128 and a batch size of 8. Two NVIDIA TITAN RTX graphics cards were used for training. In the experiment, the number of Transformer blocks λ was set to 4. The optimizer used in this paper is Adam, with all parameters at default values. The initial learning rate and weight decay for model training were 1e-4 and 1e-5, respectively, and the experimental dataset was divided into a training and the testing set uses a 6:4 ratio. (7,200 sheets in the Tongji dataset are used as the training set and 4,800 sheets are used as the testing; 360 sheets in the Poly-U dataset are used as the training set and 240 sheets are used as the testing).

### 4.2. Comparative experiments

**Figure 5:**
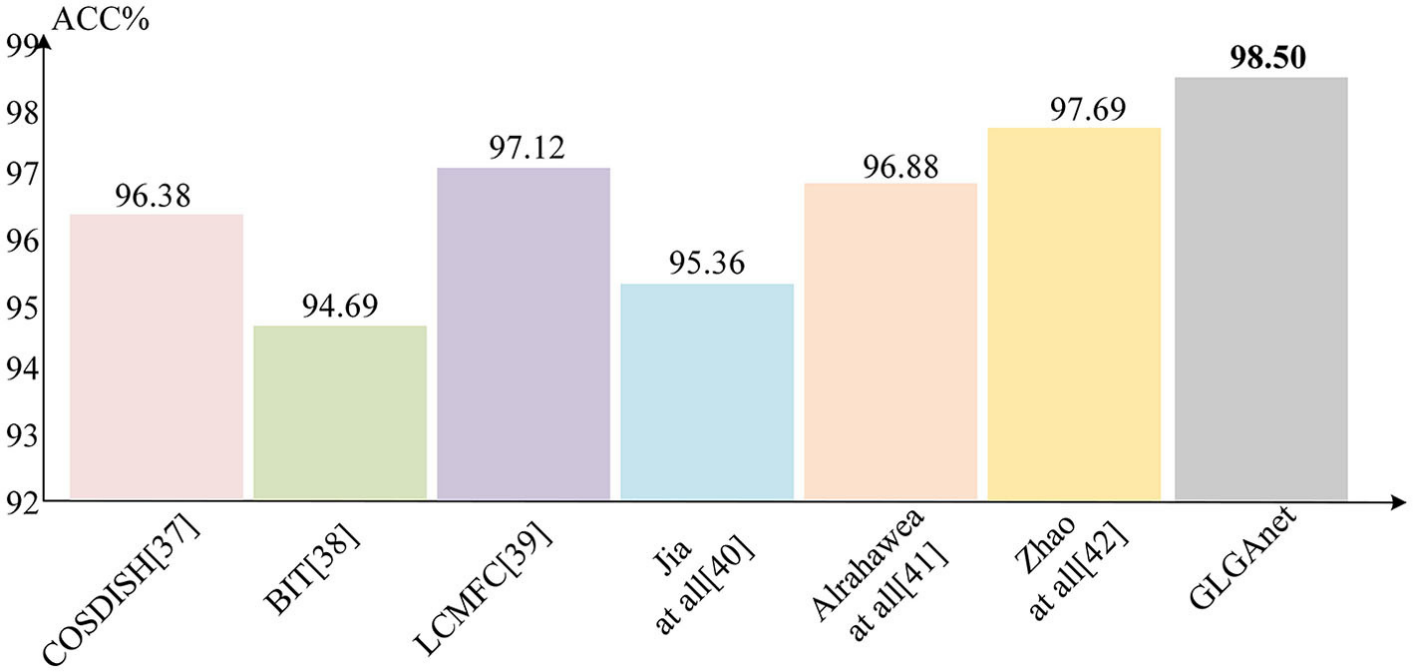
Comparison of the correct recognition rates of different methods on the Tongji dataset.

**Figure 6:**
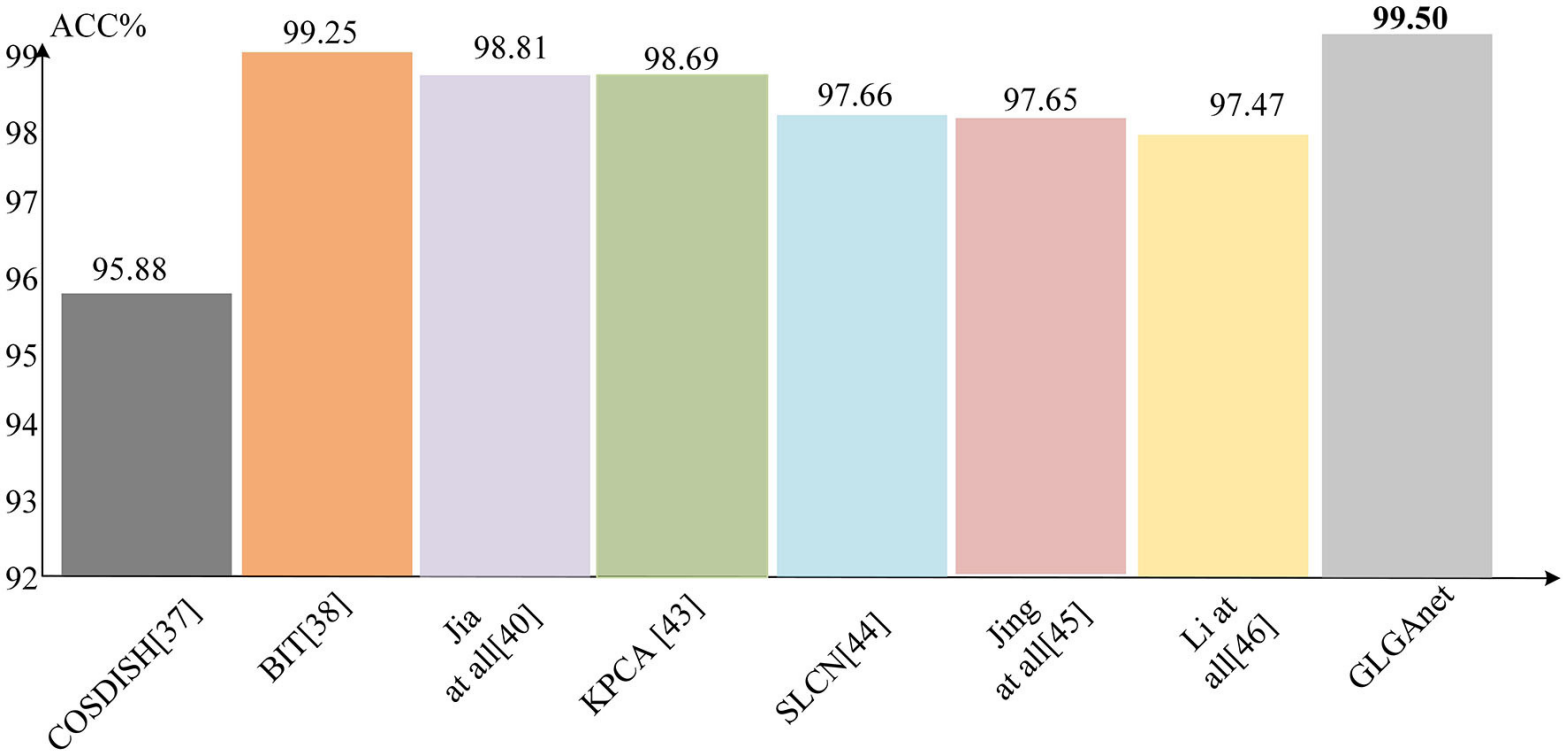
Comparison of the correct recognition rates of different methods on the Poly-U dataset.

To validate the effectiveness of the proposed method in palmprint recognition, several state-of-the-art recognition methods that were tested on the Tongji palmprint dataset and the Poly-U palmprint dataset benchmarks are compared. This includes the proposed traditional descriptor-based palmprint recognition methods (Fei et al., 2020c, 2022; Jia et al., 2020; Zhou et al., 2020; Kusban, 2021; Li et al., 2022; Zhao et al., 2022) and the deep learning-based palmprint recognition methods (Alrahawe et al., 2021; Jia et al., 2021; Jing et al., 2021; Fei et al., 2022). A brief description of these methods is given in Table 1. The source codes of many existing palmprint recognition methods are not publicly available, and to avoid biases introduced when models are retrained, publications are directly referred to obtain the results of the corresponding methods. This is a common approach in palmprint recognition research. The recognition accuracy results of the different methods for the Tongji University palmprint dataset and the Hong Kong Polytechnic University palmprint dataset are shown in Figures 5, 6, respectively. The method with the highest recognition accuracy in each dataset is bolded in the corresponding bar.

**Table 1:** Methods used for performance comparison in experiments.

**Methods**	**Description**
COSDISH (2020), Jia et al. (2020)	A discrete supervised hashing method based on column sampling is proposed for palmprint recognition.
BIT (2020), Zhou et al. (2020)	A biologically based palmprint recognition transform feature extractor (BIT) is proposed.
LCMFC (2020), Fei et al. (2020c)	It proposes a multi-feature learning method to extract directional and textural features of palmprint for palmprint recognition.
Jia et al. (2021)	A new deep palmprint hash recognition method is proposed.
Alrahawe et al. (2021)	A contactless biometric system for palmprint recognition based on convolutional neural network (ConvNet) was designed.
KPCA (2021), Kusban (2021)	It designs a palmprint recognition method using Gabor technique and kernel principal component analysis.
Jing et al. (2021)	A supervised group sparse regularizer is designed to optimize the localization and group sparsity of extracted palmprint features
Zhao et al. (2022)	It proposes a new multi-view discriminative palmprint recognition method based on double cohesive learning.
SLCN (2022), Fei et al. (2022)	It proposes a collaborative palmprint-specific binary feature learning method and a network consisting of a single convolutional layer.
Li et al. (2022)	A method for localized triple pattern recognition of palmprints based on line features is proposed.

Table 2 shows the correct recognition rates of the different methods on the two datasets. According to the results in Table 2, it is clear that the proposed method achieves high performance on both datasets compared with the traditional and deep learning based methods in the existing work. According to the results in the table that the proposed method performs better on the PolyU dataset because of its higher requirement for image alignment accuracy during the image acquisition phase. On the tongji dataset, in addition to the excellent performance of the proposed method in this paper, the next best method is a new multi-view discriminative palmprint recognition method based on double cohesive learning proposed by Zhao et al. (2022), which improves the palmprint recognition performance by using palmprint features from multiple views presented in a low-dimensional subspace. The next best performer compared with the proposed method on the PolyU dataset is a biologically based transform feature extractor (BIT) for palmprint recognition proposed by Zhou et al. (2020). This method recognizes palmprint by simulating the visual perception mechanism of simple cells, which is computationally complex, not robust and cannot be directly applied to palmprint. The proposed method achieves stable recognition performance by controlling the transfer of features through a gating mechanism.

**Table 2:** Comparison of recognition accuracy of different methods in PolyU and Tongji data set.

**Methods**	**Tongji**	**PolyU**
COSDISH (2020), Jia et al. (2020)	96.38	95.88
BIT (2020), Zhou et al. (2020)	94.69	99.25
LCMFC (2020), Fei et al. (2020c)	97.12	–
Jia et al. (2021)	95.36	98.81
Alrahawe et al. (2021)	96.88	–
Jing et al. (2021)	–	97.65
KPCA (2021), Kusban (2021)	–	98.81
SLCN (2022), Fei et al. (2022)	–	97.66
Zhao et al. (2022)	97.69	–
Li et al. (2022)	–	97.47
**GLGAnet**	**98.50**	**99.50**

Bold value indicates the best experimental results.

According to the results reported in Figures 5, 6, the method proposed in this paper obtained a more competitive performance compared with other methods. Specifically, on the Tongji University palmprint dataset, the recognition accuracy of the method proposed in this paper was 98.5%, outperforming other methods by 2.12, 3.81, 1.38, 3.14, 1.62, and 0.81%, respectively. On the Hong Kong Polytechnic University palmprint dataset, achieved 99.8% recognition rate, outperforming other methods by 3.62, 0.25, 0.69, 0.81, 1.84, 1.85, and 2.03%, respectively. On the Tongji University palmprint dataset, the existing methods of convolutional neural networks were not effective for palmprint recognition. For example, Jia et al. (2021) used a deep palmprint hash recognition method which neglected to extract the global features of palmprints and obtained the features of palmprints through an ordinary convolutional neural network. However, the ordinary convolutional neural network was limited by the size of the perceptual field and could not achieve good results even when using a hash layer and multiple loss functions to jointly supervise the training process. Alrahawe et al. (2021) designed a palmprint recognition system based on convolutional neural network, which is limited by the size. The designed convolutional neural network can only extract some shallow information and cannot achieve high recognition results. On the PolyU dataset, some researchers have designed convolutional neural networks for palmprint recognition. For example, a collaborative palmprint-specific binary feature learning method and a compact network consisting of a single convolutional layer were proposed by Fei et al. (2022). The palmprint feature learning method in this method uses a feature projection function, which loses some information of the palmprint and cannot achieve good recognition results. In contrast, the proposed method uses a deep convolutional neural network to extract local features of the palmprint image in separate channels. The global features of the palmprint are then obtained using a Res-Vit structure based on a self-attentive mechanism, allowing the designed network to avoid the shortcomings of the above study. It is possible to model and extract not only the local but also the global information of the palmprint. In particular, the proposed method also designs a gating mechanism for the selection control of the extracted features according to the different features extracted by the deep convolutional neural network and Res-Vit, making the palmprint recognition the best possible.

### 4.3. Ablation experiments

To further validate the importance and practical contribution of the backbone network and the designed modules (including the gating mechanism) used in this paper, relevant ablation experiments were conducted.

In this experiment, GLGANet backbone is used as the baseline model and then its effectiveness is verified by adding different gating mechanisms one by one. Specifically, the performance of the following five models is compared: GLGANet backbone: a good pre-trained model for palmprint recognition using the GLGANet backbone network, which is the baseline model. GLGANet backbone+G1: the first gating mechanism is added on top of the GLNet backbone network to control the features extracted by the first deep convolutional layer. GLGANet backbone+G1+G2: A second gating mechanism is added to GLGANet+G1 to control the features extracted by the first and second deep convolutional layers. GLGANet backbone+G1+G2+G3+G4:On top of the above model, a fourth gating mechanism is added to control the global features extracted by the second Transformer structure to a deeper level of the palmprint. Full model: The proposed full model (GLGANet +G1+G2+G3+G4+G5).

A comparison of GLGANet backbone and GLGANet backbone+G1 demonstrates the effectiveness of the gating mechanism used in GLGANet backbone to control the selection of the local features extracted from the first depth convolutional layer for palm prints. Comparison of GLGANet backbone+G1 and GLGANet backbone+G1+G2 shows that the gating mechanism is effective in controlling the selection of features extracted from the deep convolutional layer. Comparing GLGANet backbone+G1+G2 with GLGANet backbone+G1+G2+G3 and GLGANet backbone+G1+G2+G3+G4, it verifies that the gating mechanism is effective in controlling the local features of palmprints extracted from the depth convolution layer. It also has a control effect on the global features of the palmprint extracted by the Transformer structure. Finally, by comparing GLGANet backbone+G1+G2+G3+G4 with the full model, the effectiveness of the gating mechanism in controlling the local features and the fusion of the local and global features of the palmprint by the adaptive convolution fusion module is verified.

Table 3 lists the objective performance exhibited by the different models. Each of the gating mechanisms described above has improved the correctness of the palmprint recognition results. The results did not improve when two gating mechanisms were added. The reason is that the features extracted by adding the first two gating mechanisms were fused with the layer-level features extracted by the backbone network and then convolved and pooled several times, losing some key information. Starting with the addition of the third gating mechanism, the results were significantly improved. The recognition results are more obvious with the addition of the last five gating mechanisms and the adaptive convolution module.

**Table 3:** The results of the ablation experiment.

**Models**	**Percentage of results correct (*%*)**
GLGANet backbone	98.00
GLGANet backbone+g1	98.40
GLGANet backbone+g1+g2	98.80
GLGANet backbone+g1+g2+g3	99.00
GLGANet backbone+g1+g2+g3+g4	99.20
GLGANet backbone+g1+g2+g3+g4+g5	**99.50**

Bold value indicates the best experimental results.

**Figure 7:**
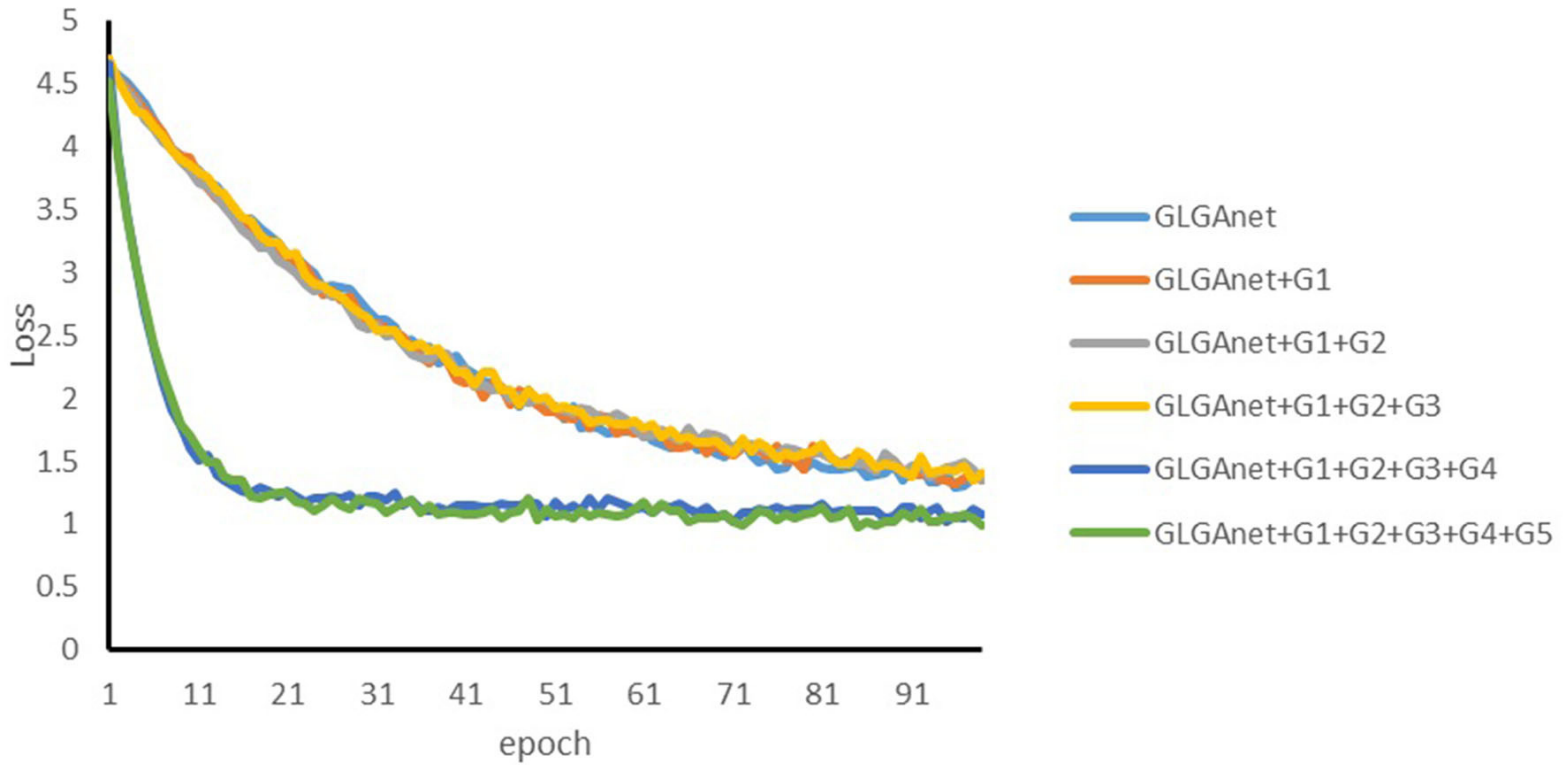
Convergence of the loss function during the training of different models.

Figure 7 shows the convergence of the loss function during the training of different models. As shown in Figure 7, the proposed method does not achieve the best result when the first three gating mechanisms are added, which also corresponds to the results of the ablation experiments. (the effect is significantly improved when the third gating mechanism is added). As the number of gating mechanisms increases, the convergence of the loss function also verifies the effectiveness of the gating mechanisms. In particular, the loss function converges most significantly and the best recognition is achieved when all five gating mechanisms are added.

## 5. Summary

This paper proposes a framework for palmprint recognition based on CNN and Transformer. The method extracts the local information of palmprint by using CNN and the global contextual information of palmprint by Transformer. In addition, a gating mechanism is designed based on the difference in the features extracted by the CNN and Transformer, and the gating mechanism is used to control the selection of the extracted local and global features. In addition, an adaptive convolutional fusion module is designed to effectively fuse the features obtained by the gating mechanism with those of the backbone network through single or multiple convolutions. Experimental results verify the effectiveness of the key components extracted by the proposed method. In addition, the method achieved better performance in benchmark tests on the Tongji palmprint dataset and Poly-U dataset compared with state-of-the-art methods. Future work may focus on exploring the use of fewer gating mechanisms to achieve high accuracy in palmprint recognition and extending the proposed method to other biometric recognition problems.

## Data availability statement

The datasets presented in this study can be found in online repositories. The names of the repository/repositories and accession number(s) can be found at: https://cslinzhang.github.io/ContactlessPalm/.

## Author contributions

All authors listed have made a substantial, direct, and intellectual contribution to the work and approved it for publication.
